# Glanzmann Thrombasthenia in Pakistani Patients: Identification of 7 Novel Pathogenic Variants in the Fibrinogen Receptor αIIbβ3

**DOI:** 10.3390/cells12020213

**Published:** 2023-01-04

**Authors:** Muhammad Younus Jamal Siddiqi, Doris Boeckelmann, Arshi Naz, Ayisha Imran, Shariq Ahmed, Akbar Najmuddin, Barbara Zieger

**Affiliations:** 1 National Institute of Blood Disease and Bone Marrow Transplantation, Karachi 75300, Pakistan; 2 Baqai Institute of Hematology, Baqai Medical University, Karachi 75340, Pakistan; 3 Department of Pediatrics and Adolescent Medicine, Division of Pediatric Hematology and Oncology, Faculty of Medicine, Medical Center–University of Freiburg, 79098 Freiburg, Germany; 4 Department of Pathology, Liaquat University of Medical and Health Sciences, Jamshoro 76090, Pakistan; 5 Chughtai Lab Lahore, Lahore 54000, Pakistan; 6 Fatimid Foundation, Karachi 74800, Pakistan

**Keywords:** Glanzmann thrombasthenia, inherited platelet disorder, consanguineous, ITGA2B, ITGB3, Pakistan

## Abstract

Glanzmann thrombasthenia (GT) is a rare autosomal recessive inherited platelet disorder occurring frequently in populations with high incidence of consanguineous marriages. GT is characterized by quantitative and/or qualitative defect of the platelet αIIbβ3 (GPIIb/IIIa) receptor caused by pathogenic variants of the encoding genes: *ITGA2B* and *ITGB3*. Patients present with a moderate to severe bleeding tendency with normal platelet count. Platelets show reduced/absent aggregation for all agonists except ristocetin in light transmission aggregometry and reduced/absent αIIbβ3 expression in flow cytometry (FC). In this study, we investigated a cohort of 20 Pakistani patients and 2 families collected from the National Institute of Blood Disease, Karachi and Chughtai’s Lab, Lahore. Platelet aggregation studies, FC (platelet CD41, CD61, CD42a, CD42b) and direct sequencing of the candidate genes were performed. All patients showed altered platelet aggregation, but normal agglutination after stimulation with ristocetin. Absent/reduced αIIbβ3 receptor expression was present in the platelets of 16 patients, in 4 patients expression was borderline/normal. Candidate gene sequencing identified pathogenic/likely pathogenic variants in 15 patients. Seven variants are novel. One patient with absent receptor expression remained without genetic finding. 13 (86.7%) of 15 patients stated consanguinity reflected by homozygosity finding in 14 (93.3%) patients.

## 1. Introduction

Among the inherited platelet disorders (IPD) Glanzmann thrombasthenia (GT) [Online Mendelian Inheritance in Man (OMIM) #273800] is the most commonly presented disease [1]. Eduard Glanzmann, a Swiss pediatrician, was the first to describe the disease in 1918 as “hereditary hemorrhagic thrombasthenia” [2]. It has an estimated worldwide prevalence of 1 in 1 million but occurs in higher frequency in ethnic populations where intermarriages are common, like in Pakistan [3]. GT is a moderate to severe hemorrhagic IPD, characterized by recurrent mucocutaneous bleeding. The patients present with mucosal gum bleeding, purpuric skin rash, epistaxis, and menorrhagia. Bleeding episodes are usually not fatal; however, life-threatening bleeding can occur in case of surgery in mucocutaneous regions. Laboratory studies reveal normal platelet count and morphology whereas the bleeding time is prolonged [1]. PT, APTT and fibrinogen levels are normal. Light transmission platelet aggregometry (LTA) reveals impaired platelet aggregation after stimulation with collagen and other agonists. Platelet agglutination after stimulation with ristocetin often reaches the normal maximum range. Flow cytometry (CD41, CD61) demonstrates reduced or absent integrin αIIbβ3 receptor expression [4,5]. Depending on the αIIbβ3 expression GT may be classified as type 1 (<5% of normal αIIbβ3 level) or type 2 (5–25%). Furthermore, the receptor may be normally expressed; however, functionally impaired resulting in defective binding of fibrinogen.

Inheritance is mainly autosomal recessive. Pathogenic variants in the receptor-coding genes *ITGA2B* and *ITGB3* (located on the long arm of chromosome 17 (q21–22) have been identified as disease causing. *ITGA2B* spans 17 kilobases (kb) and comprises 30 exons; *ITGB3* (46 kb) has 15 exons. Pathogenic variants lead to qualitative or quantitative abnormalities regarding the expression of the αIIbβ3 receptor. Activation of the receptor enables fibrinogen binding which leads to conformational changes in the receptor complex [6,7]. During recent years, multiple pathogenic variants have been identified for both genes and documented in the Human gene mutation database (HGMD) and in disease specific database from the Medical College of Wisconsin (https://glanzmann.mcw.edu/). Management of patients include transfusion of platelet concentrates (prior vaccination is highly desired). HLA matched transfusion is reserved for patients with severe bleeding tendency. Localized bleeding, (epistaxis or gum bleeding), can be managed with conservative local measures such as application of gauze pads and/or fibrin sealants containing fibrinogen, thrombin, factor XIII and aprotinin, or fibrin-coated collagen fleece.

The study comprised of two phases. The first phase encompassed 429 patients, (250 males and 179 females) patients with bleeding disorders. 211 [49.1%] were diagnosed with an autosomal bleeding disorder (ARBD) [8]. In the second phase a cohort of 26 patients previously identified with a suspected platelet functional disorder (PFD) from the first phase, were recruited from tertiary health care centers in Sindh and Punjab. Informed and written consent was obtained as per declaration of Helsinki. 6 of these 26 patients were diagnosed with Bernard Soulier syndrome [9]. In this part of the study, we performed platelet aggregometry, flow cytometry and candidate gene sequencing for 20 patients to identify the cause of the bleeding disorder and families of 2 patients were also screened to identify their molecular characteristics.

## 2. Patients and Methods

This cross-sectional study was approved by the institutional review board and ethical committee of National Institute of Blood Diseases and Bone Marrow Transplantation (NIBD), Karachi, and by the ethics committee of the University Hospital Freiburg, Germany. Bleeding assessment was done using the bleeding score by Tosetto et al. [10].

### 2.1. Sample Collection and Biochemical Analysis

2.7 mL of venous blood sample was collected from each patient into trisodium citrate 0.109 M 3.2% vacutainer tube. 3 mL blood was collected in EDTA vacutainer tubes for platelet count and DNA extraction. First-line coagulation profile, including PT and APTT were performed on all samples, fibrinogen levels were measured by Clauss method. Platelet counts were performed on Sysmex XN 1000. Peripheral blood smear was observed under the microscope for platelet morphology.

### 2.2. Platelet Aggregregometry Analyses

Platelet aggregometry studies were performed using standard light transmission aggregometer (Helena Aggram, aggregation remote analyzer module, Beaumont, TX, USA) after stimulation with different agonists (ADP, 2.25 µM; epinephrine, 5 µM; collagen, 4 µg/mL; ristocetin, 0.5 mg/mL; arachidonic acid, 500 µg/mL).

### 2.3. Flow Cytometry Analyses

Receptor expression was investigated using flow cytometry (BD FACSCalibur) and different monoclonal antibodies i.e., anti-CD42a fluorescein isothiocyanate (FITC, GPIX complex, clone Beb1; Becton Dickinson (BD), San Jose, CA, USA), anti-CD42b (GPIbα, clone SZ2; Immunotech, Marseille, France), anti-CD41 phycoerythrin (PE) (GPIIb, αIIb integrin, clone P2; Immunotech), and anti-CD61 peridinin chlorophyll protein (PerCP, GPIIIa, β 3 integrin, clone RUU-PL 7F12; BD). Data were analysed using CELL QUEST PRO software (BD FACStation software©2007, Becton, Dickinson, and Company, Dr, Franklin Lakes, NJ, USA).

### 2.4. Extraction, Amplification and Sequencing of Genomic DNA

Genomic DNA was extracted from peripheral whole blood using QiAmp DNA Blood mini-Kit (Qiagen^®^). DNA concentration and purity were measured by Qubit^®^ 2.0 flourometer (Life Technology^®^, CA, USA). The candidate genes *ITGA2B* and *ITGB3* were amplified by exon specific polymerase chain reaction (PCR) with primers sets, which covered 45 exons. 25–100 ng of genomic DNA was used as template along with primer concentration of 20 pM, 200 µM dNTPs, 1 mM MgCl2 and 1 unit Dream Taq DNA polymerase (thermoscientific^®^, Waltham, MA, USA) in 25 uL reaction buffer. PCR conditions were: Initial denaturation at 95 °C for 5 min, then further denaturation at 95 °C for 30 s, annealing at 61 °C for 30 s, extension at 72 °C for 30 s followed by 5 cycles and continued denaturation at 95 °C for 30 s, annealing at 58 °C for 30 s, extension at 72 °C for 30 s followed by 28 cycles and final extension at 72 °C for 10 min. PCR products were separated on 2% agarose gels and visualized by fluorescence under UV light. Amplified products were sequenced by chain termination method using a fluorescence-labeled dideoxynucleotides ABI big dye terminator kit v.3.1 according to protocol. Chromatograms were aligned using CodonCode Sequence Assembly and Alignment Software (Version 7.1.2, Natick, MA, USA.). Variants were named according to HGVS nomenclature and analyzed using supporting software ALAMUT^®^ VISUAL. Occurrence in variant databases (dbSNP, Exome Variant Server (EVS), Exome Aggregation Consortium (ExAC), gnomAD) and in locus specific databases (HGMD, mcw.GT database), conservation status and in silico pathogenic prediction was obtained. Variants not listed in databases such as HGMD public or in GT specific database from the Medical College of Wisconsin (former Sinai Central db) were considered novel (access on 01 February 2022). Pathogenic, likely pathogenic variants, or VUS with deleterious in silico prediction were confirmed in a second independent PCR followed by Sanger sequencing. Because of lack of material, a second independent PCR could not be performed for pt.12. Family genotyping was performed with available samples. Data was analyzed by SPSS (version 16, Chicago, IL, USA) using descriptive statistics.

## 3. Results

Of the 20 patients investigated 12 patients were male (46.1%) and 8 (30.7%) females. 88.4% patients were born to parents with consanguineous marriages. 12 index patients were unrelated, 8 were affected siblings from 4 families.

All patients showed normal values for first line coagulation tests (PT and APTT), fibrinogen (data not shown). 18 patients presented with normal platelet count and morphology, 2 patients (pt.7 and 12) with a platelet count of 124,000 and 128,000 per microliter, respectively. Light transmission aggregometry showed impaired aggregation pattern with ADP, collagen, epinephrine, and arachidonic acid and normal ristocetin response, hinting among others to GT. Flow cytometry analyses showed absent or reduced expression of CD41 (αIIb) and CD61 (β3) for 16 patients. 4 patients showed normal or borderline expression values for CD41 (ranging from 70 to 81 MFI) and for CD63 (ranging from 65 to 85 MFI), respectively). Results are summarized in Table 1. All patients showed normal expression of CD42a and CD42b.

Candidate gene analyses identified pathogenic, likely pathogenic or variants of uncertain significance with deleterious in silico prediction in 15 patients with absent or reduced receptor expression: 6 *novel* variants in *ITGA2B* and 1 *novel* variant in *ITGB3* (Figure 1). Direct sequencing chromatograms are displayed in supplementary (Figures S1–S14).

8 patients carried homozygous variants of all types (6 nsSNV, 1 STOP, 1 duplication, 2 canonical splice-sites) in *ITGA2B*, one patient (pt.6) had two different mutations, one was already reported and the other one a novel nsSNV in *ITGA2B* (Table 2).

Additional to the most likely disease-causing homozygous splice variant (c.188 + 1G > A) in *ITGA2B,* patient 1 carried a heterozygous private or exceedingly rare variant in *ITGB3* (c.20C > G, p.Pro7Arg). This variant is located in the signal peptide sequence of ß3 with benign in silico prediction (not listed in table).

In *ITGB3* we identified the c.422A > G variant homozygous in 2 related patients (pt.10 and pt.11) and one unrelated (pt.12). Two related patients (pt.13 and 14) are homozygous carriers of the already reported c.428T > G variant, whereas another unrelated patient (pt.15) showed an alteration from T > C at the same position. The resulting likely pathogenic amino acid alteration (p.Leu143Ser) is not reported so far (Table 3).

In one patient (pt.16) with absent αIIbβ3 expression we could not identify a disease-causing variant in the candidate genes for GT. In the group of 4 patients with altered LTA but normal or borderline receptor expression, we identified in one patient (pt.17) a heterozygous variant (c.197T > G, p.Leu66Arg) of divergent classification (unknown significance/likely benign) in *ITGB3*. For the other 3 patients we could also not identify a disease-causing variant in the candidate genes for GT.

Genetic analysis of the parents of the siblings pt.4 and pt.5 showed that both parents are heterozygous carrier for the *ITGA2B* c.857T > A variant. Investigating the family of patient 8 revealed the mother as a heterozygous carrier of the nonsense mutation in exon 14 of the *ITGA2B* gene, resulting in a premature stop codon. We also found the same heterozygous defect in the patient´s first sibling whereas the second sibling presented as wild type. The father was already deceased; no DNA was available from him. All heterozygous carriers showed only minor reduction of receptor expression and were phenotypically not affected (Table 4).

## 4. Discussion

Although prevalence of GT is rare worldwide, it is hypothesized that in south Asia GT has a higher prevalence because of the consanguinity. Using candidate gene analysis, we molecular genetically confirmed autosomal recessive Glanzmann Thrombasthenia for 15 patients showing absent or reduced αIIbβ3 expression. 14 patients were homozygous for the variants identified and out of these 12 had consanguineously married parents.

### 4.1. Novel Variants in ITGA2B Classified as Pathogenic (Class 5) Because of Serious Consequences

We homozygously identified 4 novel variants which lead to serious consequences regarding the αIIb subunit: one nonsense mutation (c.1423C > T, p.Gln475*), one frameshift mutation (c.3001_3010dup, p.Val1004Alafs*35) and 2 canonical splice site mutations c.188 + 1G > A; c.575−2A > T). We demonstrated that heterozygous c.1423C > T family members were phenotypically not affected and showed only minor reduction of αIIbβ3 expression. The patient (pt.9) with the frameshift mutation (c.3001_3010dup) suffers from severe bleeding symptoms. The alteration is located quite at the end of the αIIb subunit in the C-terminal region and flow cytometry analysis showed an absent expression of CD 41 and CD 61. A complete absence of αIIbβ3 has been also reported for a compound heterozygous GT patient with a similar frameshift alternating variant (c.3016insG) in this cytoplasmic region together with a premature STOP codon [16]. The canonical splice site mutation c.188 + 1G > A (pt.1) is located in the donor splice site of intron 1 resulting most likely in skipping of exon 1. A homozygous variant affecting the last nucleotide in exon 1 (c.188G > A) causes most likely a similar splicing error and has already been reported [17]. The canonical splice site mutation c.575-2A > T (pt.2) is located in the acceptor splice site of intron 4 and results most likely in skipping of exon 5. Both patients with these splice site mutations had absent or severely reduced αIIbβ3 expression. The loss of receptor expression seen in all patients with these novel pathogenic variants are due to a premature STOP codon, to a frameshift/protein truncation, and to splice defects.

### 4.2. Novel Variants in ITGA2B Classified as likely Pathogenic or as Variant of Uncertain Significance with Pathogenic Prediction

The novel nsSNV c.1355T > G (pt.7, rs775251867, MAF gnomAD ALL: 0.0004%) in exon 13 leads to substitution of leucine by arginine (p. Leu452Arg) and is classified deleterious by in silico pathogenic prediction (SIFT, MutationTaster, PolyPhen2). The alteration affects a highly conserved amino acid in the alpha beta propeller region of the alpha chain. The patient (pt.7) homozygous with this variant presented with a high bleeding score of 10 and a history of frequent transfusions. There was no CD41/CD61 expression detectable by platelet flow cytometry. Patient 6 is compound heterozygous with two missense variants both located in the beta propeller chain of the alpha integrin involving the FG-GAP repeat, which folds the beta propeller chain with the alpha chain. The first variant (c.989A > T; p.Asn330Ile) has a deleterious in silico prediction (SIFT, MutationTaster, PolyPhen2), the second (c.1028T > C; p.Leu343Pro) has already been reported. 

### 4.3. Variants in ITGA2B Which Have Been already Reported

Patient 3 is homozygous for c.659A > G (rs1355838837), resulting in amino acid alteration Tyr220Cys. The nucleotide at position c.659 is weakly and Tyr220 is moderately conserved, however, there is a large physiochemical difference between Tyr and Cys (Grantham dist. 194 (0–215)). The alteration is located in the beta propeller region of the alpha integrin with deleterious pathogenicity prediction. In our study, patient 3 reported mild to moderate bleeding episodes; platelet αIIbβ3 expression was moderately decreased. The Missense alteration c.659A > G is compound heterozygous together with a slice-site mutation reported for a GT type II patient from China [11]. This patient presented with αIIbβ3 expression between 10–20%. In two siblings of our study (pt.4 and pt.5) we identified a homozygous variant (c.857T > A, p. Val286Asp) in exon 9 of *ITGA2B*. Val286Asp (V255D in mature protein) is a well-studied mutation located in the β-propeller region of αIIb. Substitution of the amino acid forms an additional polar structure in the hydrophobic environment, resulting in destabilization of the integrin. The water molecules then enter and form H-bonds with oxygen atoms or NH2-groups of constituent amino acids and therefore disrupt the structure [12].

### 4.4. Variants in ITGB3 

In two siblings (pt.10 and 11) and in another unrelated patient (pt.12) belonging to the same geographical region we identified a homozygous pathogenic variant (c.422A > G, p. Tyr141Cys) already reported in Indian patients [13]. In pt.10 an additional heterozygous variant (c.383T > G, p.Ile128Ser) was found. The physiochemical difference between these two amino acids located at the N-terminus is moderate (Grantham distance: 142 (0–215)), serine is polar uncharged instead of the hydrophobic isoleucine. The wildtype amino acid is only weakly conserved in 14 species. The altered amino acids leucine (mouse) and valine (cat, cow, chicken) instead of wildtype isoleucine are all hydrophobic. Because of divergent in silico pathogenicity interpretations (SIFT, PolyPhen2: deleterious and probably damaging; MutationTaster: Polymorphism) we classified the variant as class 3 (uncertain significance). 

Leu143Trp (c.428T > G, rs121918452) identified in the siblings pt.13 and pt.14 affects the protein domain of the beta subunit in the N-terminal region. This results in the intracellular retention of misfolded αIIbβ3 heterodimers and has been described in a Pakistani child whose platelets express less than 10% of the total amount of αIIbβ3 [14] and in the Indian population [15]. Peretz et al suggest that this variant is an Indian founder mutation [15]. Interestingly, the patient who was investigated in this study has parents who migrated from India to Pakistan. Grantham score between the wild type amino acid leucine (hydrophobic) and the altered tryptophan (hydrophobic) is 61 (0–215). Leu143Ser (c.428T > C) identified in pt.15, an alteration at the same position has not been described so far and therefore, we classified this variant as likely pathogenic. Grantham score between the wild-type amino acid leucine (hydrophobic) and serine (polar uncharged) is 145 (0–215) and therefore higher than in exchange with tryptophan. In the patient reported here the expression of CD61 (β3) was severely reduced. 

Heterozygous Leu66Arg (c.197T > G, rs36080296) was identified in patient 17 who presented with normal receptor expression. This alteration is located in the N-terminal protein domain of the beta subunit, affecting the plexin-like fold. Pathogenic prediction is concordant deleterious (SIFT, MutationTaster, PolyPhen2). In 2021 the ClinGen Platelet Disorders Variant Curation Expert Panel classified the variant as VUS. Minor allele frequency in gnomAD is 0.18% (ALL). Therefore, it remains unclear if this variant may explain the patient´s mild bleeding symptoms (mainly gum bleeding) occurring rarely due to his carrier status. Normally heterozygous carriers of a pathogenic GT variant are clinically not affected. Nevertheless, there could be variants in other genes responsible for the mild phenotype of the patient.

International studies have reported 20% of GT patients with unidentified mutations [4,18]. Our genetic approach of candidate gene sequencing confirmed GT diagnosis for 15 patients of our study. However, in 1 patient with absent receptor expression, no disease related variant in the coding region or canonical splice sites of the candidate genes could be identified. The defect could also be due to larger deletions/insertions or intronic variants leading to altered splicing. A previous study has suggested that defects in the elements for regulation of transcription may be also responsible for the presentation of the disease [19].

3 patients of our study (pt.18–20) suffered from severe bleeding episodes. Platelet aggregometry studies in these patients showed impaired results hinting to GT, however, expression of αIIbβ3 was normal/borderline (flow cytometric) and a molecular defect could not be identified. To further investigate if these patients suffer from a disorder regarding the activation of the αIIbβ3 receptor, fibrinogen binding after ADP stimulation should be performed in a future analysis (flow cytometry). Alterations in the *RASGRP2-* gene leading to CalDAG-GEFI deficiency are described as functional Glanzmann, with normal expression of the receptor, however, with defective activation [20]. Alternatively, another gene could be affected which influences function of the αIIbβ3 receptor (i.e. FERMT3). For these patients next generation sequencing (NGS) may help to identify the molecular defect. Altogether we diagnosed 16 patients with GT and confirmed molecular genetically the disease for 15 patients. Seven variants identified were novel and not reported before.

## Figures and Tables

**Figure 1:**
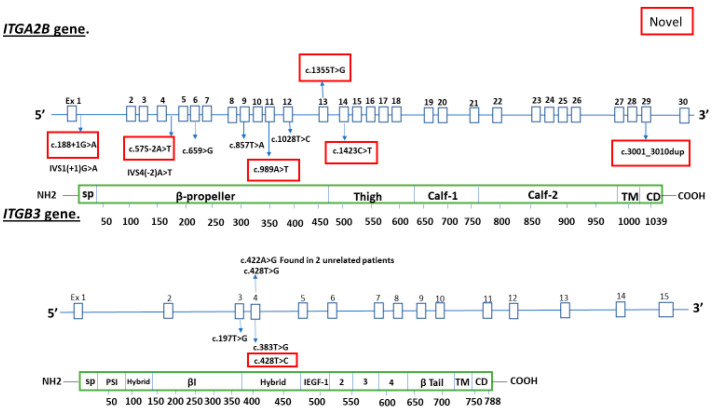
Variants detected in *ITGA2B* and *ITGB3. Novel* variants indicated in red box.

**Table 1:** Patient and platelet characterization.

ID	Age (yrs)	Gender F/M	CS	BS	Platelet Count 10^9^/L	Platelet Aggregation Studies: in % of Aggregation [Normal Max. Agg: >55%]					Flow Cytometric Analysis			
						ADP [2.25 µM]	Coll [4 µg/mL]	Epi [5 µM]	Risto [0.5 mg/mL]	AA [500 µg/mL]	CD41 (αIIb) [MFI] (56–75%)	CD61 (β3) [MFI] (57–73%)	CD42a [MFI] (53–69%)	CD42b [MFI] (52–75%)
**1**	14	M	Y	8	313	4	2	4	55	5	**0**	**0**	52	54
**2**	1.5	F	Y	7	525	2	4	3	51	3	**1**	**23**	86	73
**3**	30	M	N	9	189	4	3	3	59	2	**47**	**73**	74	74
**4_F1**	18	M	Y	8	254	3	3	5	53	3	**0**	**0**	75	62
**5_F1**	22	F	Y	11	338	4	3	5	55	5	**0**	**0**	61	54
**6**	20	M	Y	10	186	2	5	4	58	3	**4**	**9**	85	77
**7**	18	M	Y	10	124	3	2	5	60	4	**0**	**0**	83	55
**8**	15	F	Y	14	166	4	3	4	52	2	**0**	**0**	71	57
**9**	6	F	Y	14	247	5	5	5	55	4	**0**	**0**	73	69
**10_F2**	20	M	Y	10	185	5	4	5	56	4	**1**	**0**	80	61
**11_F2**	23	F	Y	11	150	3	5	4	57	3	**0**	**0**	72	62
**12**	26	M	N	7	128	2	4	4	60	4	**0**	**0**	60	67
**13_F3**	21	M	Y	7	319	3	3	3	51	5	**0**	**0**	69	75
**14_F3**	28	F	Y	12	368	3	4	3	62	5	**0**	**0**	76	55
**15**	22	M	Y	14	358	3	5	4	64	3	**51**	**17**	90	71
**16**	23	M	Y	9	128	3	4	4	55	5	**1**	**0**	77	59
**17**	32	M	Y	6	268	3	2	3	55	3	**81**	**83**	83	75
**18_F4**	3.5	M	Y	7	302	3	5	3	55	3	**88**	**85**	83	79
**19_F4**	7	F	Y	9	310	4	5	5	55	3	**76**	**73**	81	70
**20**	7	F	Y	8	297	3	3	5	55	5	**70**	**65**	78	72

F, family; Gender: F, female, M, male; CS, Consanguinity: Y, yes, N, No; BS, Bleeding score; Coll, collagen, Epi, epinephrine; Risto, ristocetin; AA, arachidonic acid; Expression of the integrin αIIbβ3 receptor measured with anti-CD41 and anti-CD61 marked in bold.

**Table 2:** Variants in *ITGA2B* (NM_000419.4).

Pt.	Exon	Allele	Nucleotide/ Amino Acid	MAF gnomAD/dbSNP rsId	In Silico PP	Remarks
1	Donor splice site of intron 1	2	c.188 + 1G > A/ Predicted change at donor site 1 bps upstream: −100%	no	MaxEnt:−100.0% NNSPLICE:−100.0% SSF:−100.0%	Novel
2	Acceptor splice site of intron 4	2	c.575–2A > T/ Predicted change at acceptor site 2 bps downstream: −100%	no	MaxEnt:−100.0% NNSPLICE:−100.0% SSF:−100.0%	Novel
3	6	2	c.659A >G/p.Tyr220Cys	MAF 0.00079%, rs1355838837	SIFT/MutTaster: D PolyPhen 2: Probably damaging	Reported compound heterozygous [11]; ClinGen Exp.#: LP
4_F, 15_F1	9	2	c.857T > A/ p.Val286Asp	no	SIFT/MutTaster: D PolyPhen 2: Possibly Damaging	Reported [12]
6	11	1	c.989A > T/ p.Asn330Ile	no	SIFT/MutTaster: D PolyPhen 2: Probably damaging	Novel
	12	1	c.1028T > C/ p.Leu343Pro	no	SIFT/MutTaster: D PolyPhen 2: Probably damaging	Reported [4]; ClinGen Exp.#: LP
7	13	2	c.1355T > G/ p.Leu452Arg	MAF 0.00079%, rs775251867	SIFT/MutTaster: D PolyPhen 2: Probably damaging	Novel
8	14	2	c.1423C > T/ p.Gln475*	no		Novel
9	29	2	c.3001_3010dup/ p.Val1004Alafs*35	no		Novel

Pt. patient; F, Family; MAF, minor allele frequency; gnomAD, Genome Aggregation Database (v2.1.1); PP, pathogenicity prediction, D, deleterious, ClinGen Exp.#, reviewed by ClinGen Platelet Disorders Variant Curation Expert Panel and classified as LP (likely pathogenic).

**Table 3:** Variants in *ITGB3* (NM_000212.2).

Pt.	Exon	Allele	Nucleotide/ Amino Acid	MAF gnomAD dbSNP rsId	In Silico PP	Remarks
10_F2	4	1	c.383T > G/p.Ile128Ser	no	SIFT: D MutTaster: 50/50 del/benign, PolyPhen2: Possibly Damaging	VUS
	4	2	c.422A > G/p.Tyr141Cys	no	SIFT/MutTaster: D PolyPhen 2: Probably damaging	Reported [13], ClinGen Exp.§: VUS
11_F2	4	2	c.422A > G/p.Tyr141Cys	no	SIFT/MutTaster: D PolyPhen 2: Probably damaging	Reported [13], ClinGen Exp.§: VUS
12	4	2	c.422A > G/p.Tyr141Cys	no	SIFT/MutTaster: D PolyPhen 2: Probably damaging	Reported [13], ClinGen Exp.§: VUS
13_F3 14_F3	4	2	c.428T > G/p.Leu143Trp	MAF 0.00397%, rs121918452	SIFT/MutTaster: D PolyPhen 2: Probably damaging	Reported [14,15], ClinGen Exp.#: LP
15	4	2	c.428T > C/p.Leu143Ser	*no*	SIFT/MutTaster: D PolyPhen 2: Probably damaging	Novel
17	3	1	c.197T > G/p.Leu66Arg	MAF 0.35293%, rs36080296	SIFT/MutTaster: D PolyPhen 2: Probably damaging	ClinGen Exp.§: VUS

Pt., patient; FID, family ID; MAF, minor allele frequency; gnomAD, Genome Aggregation Database (v2.1.1); PP, pathogenicity prediction, D, deleterious, VUS, variant of uncertain significance; ClinGen Exp.§: reviewed by ClinGen Platelet Disorders Variant Curation Expert Panel and classified as VUS (variant of uncertain significance); ClinGen Exp.#, reviewed by ClinGen Platelet Disorders Variant Curation Expert Panel and classified as LP (likely pathogenic).

**Table 4:** Family studies: Flow cytometry and molecular genetic results for two families with *ITGA2B* pathogenic variants. Patients in bold.

	Flow Cytometric Analysis					
Patient/ Proband	CD41 (αIIb) [MFI] (56–75%)	CD61 (β3) [MFI] (57–73%)	Allele	Nucleotide	Amino Acid	Remarks
**Pt.4**	**0**	**0**	**2**	c.857T > A	p.Val286Asp	Reported [12], but no phenotype description
**Pt.5**	**0**	**0**	**2**			
Father	54	63	1			
Mother	64	66	1			
**Pt.8**	**0**	**0**	**2**	c.1423C > T	p.Gln475*	Novel
Mother	59	60	1			
Sibling 1	58	62	1			
Sibling 2	77	75	2	WT		

## Data Availability

Not applicable.

## Supplementary Materials

The following supporting information can be downloaded at: https://www.mdpi.com/article/10.3390/cells12020213/s1. Figures S1–S14: Direct sequencing chromatograms.
