# Supplementary material for: Glanzmann Thrombasthenia in Pakistani Patients: Identification of 7 Novel Pathogenic Variants in the Fibrinogen Receptor αIIbβ3
Source: Cells. 2023 Jan 4;12(2):213. doi: 10.3390/cells12020213 (PMC9856889; doi:10.3390/cells12020213)
Supplement: Supplementary file 1 [file cells-12-00213-s001.zip › cells-2066255-supplementary.pdf]

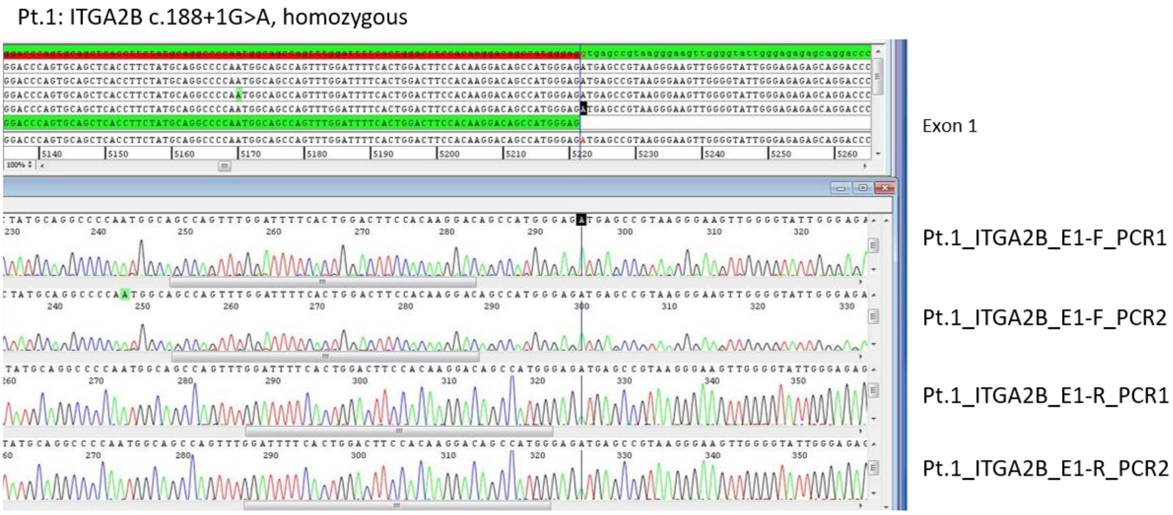

Figure S1: Chromatograms of Pt.1 showing the homozygous canonical splice variant c.188+1G>A in *ITGA2B*

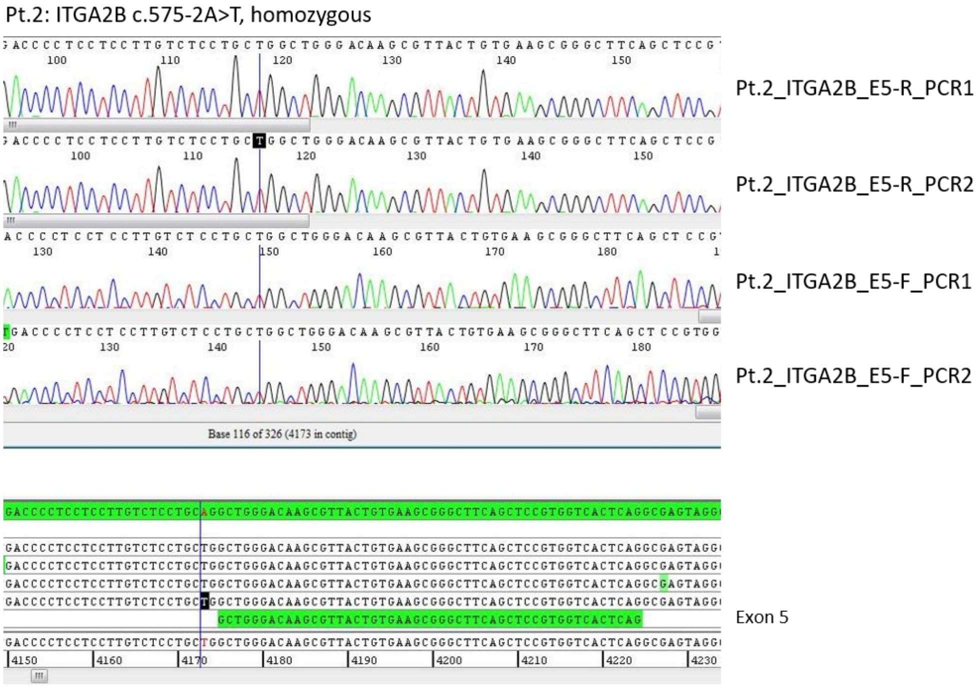

Figure S2: Chromatograms of Pt.2 showing the homozygous canonical splice variant c.575-2A>T in *ITGA2B*

Pt.3: ITGA2B c.659A>G, homozygous

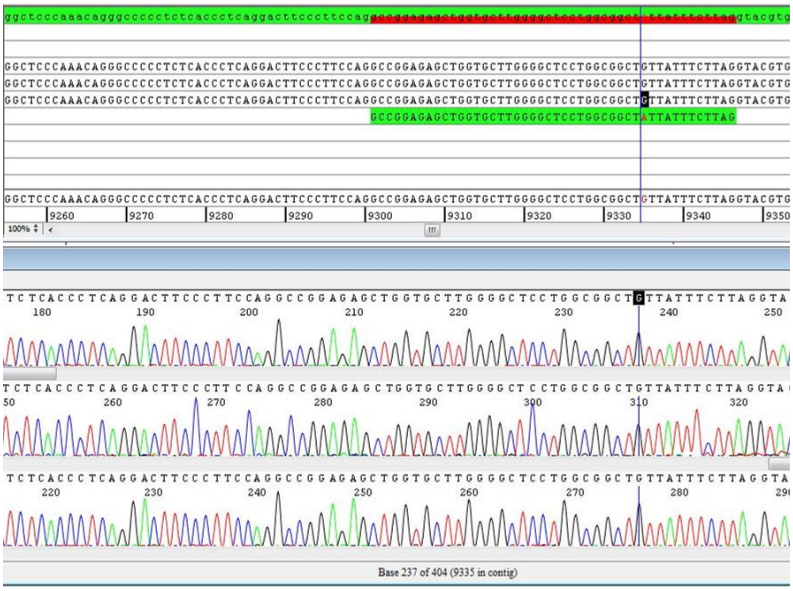

Exon 6

Pt.3_ITGA2B_E6-F_PCR1

Pt.3_ITGA2B_E6-R_PCR1

Pt.3_ITGA2B_E6-F_PCR2

Figure S3: Chromatograms of Pt.3 showing the homozygous non-synonymous variant c.659A>G (p.Tyr220Cys) in *ITGA2B*

Pt.4 and 5: ITGA2B c.857T>A; homozygous; parents heterozygous

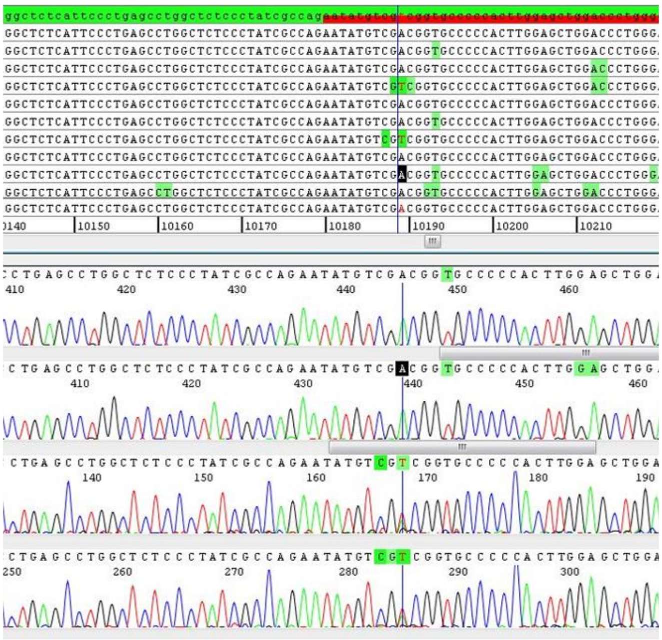

Pt.4_ITGA2B_E9-F_PCR1

Pt.5_ITGA2B_E9-F_PCR1

Mother_ITGA2B_E9-R_PCR1

Father_ITGA2B_E9-R_PCR1

Figure S4: Family 1 genotyping; chromatograms for Pt.4 and Pt.5 showing the homozygous non-synonymous variant c.857T>A (p.Val286Asp) in *ITGA2B.* The parents are heterozygous.

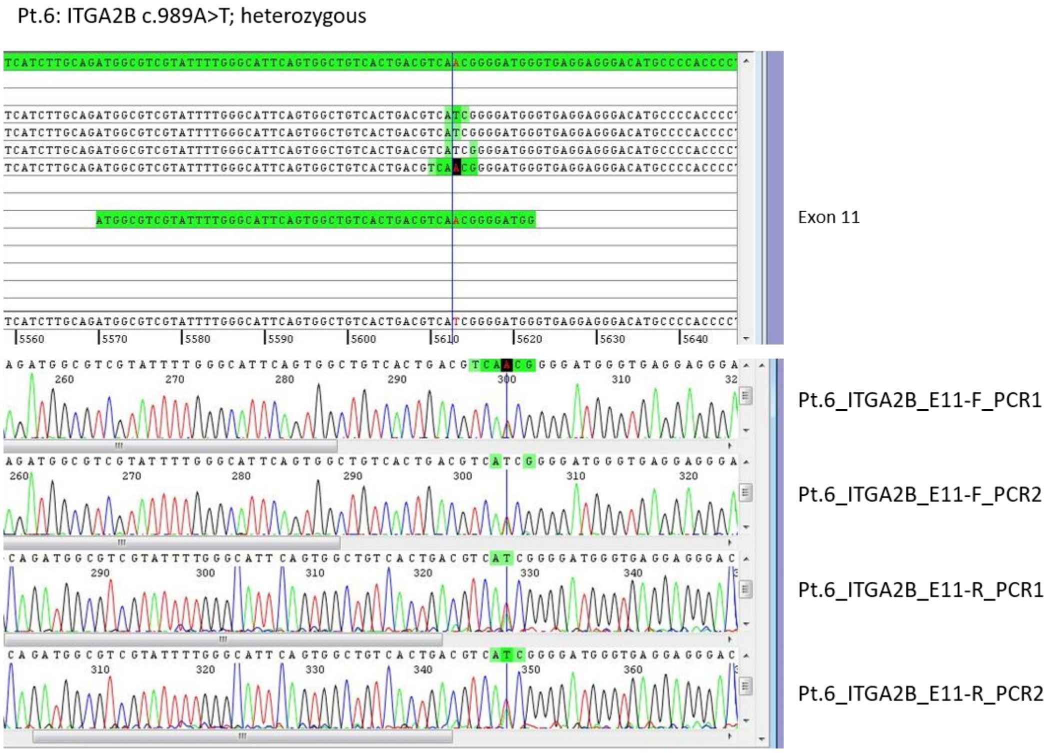

Figure S5: Chromatograms of Pt.6 showing the heterozygous non-synonymous variant c.989A>T (p.Asn330Ile) in *ITGA2B*

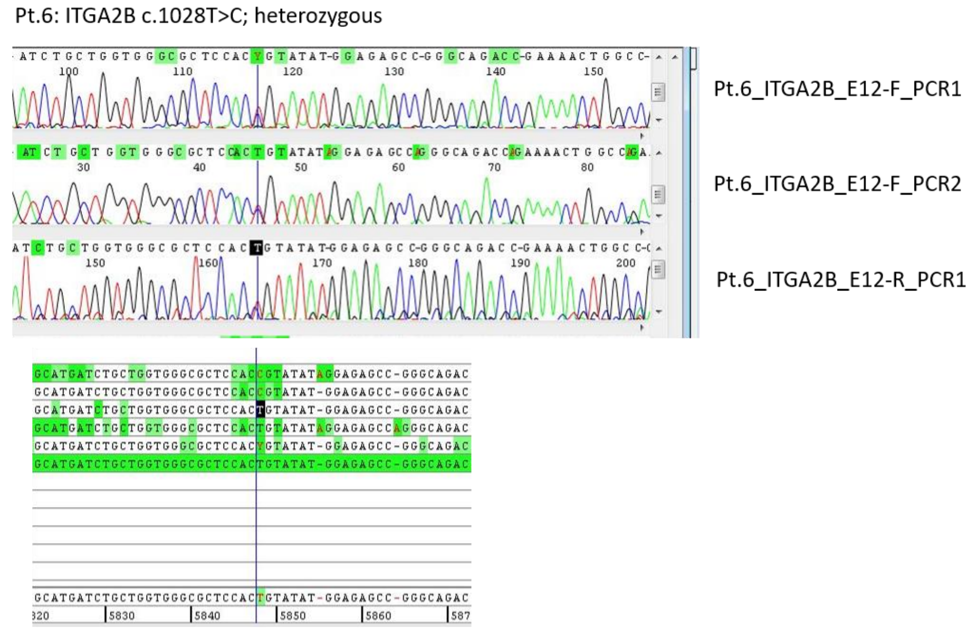

Figure S6: Chromatograms of Pt.6 showing the heterozygous non-synonymous variant c.1028T>C (p.Leu343Pro) in *ITGA2B*

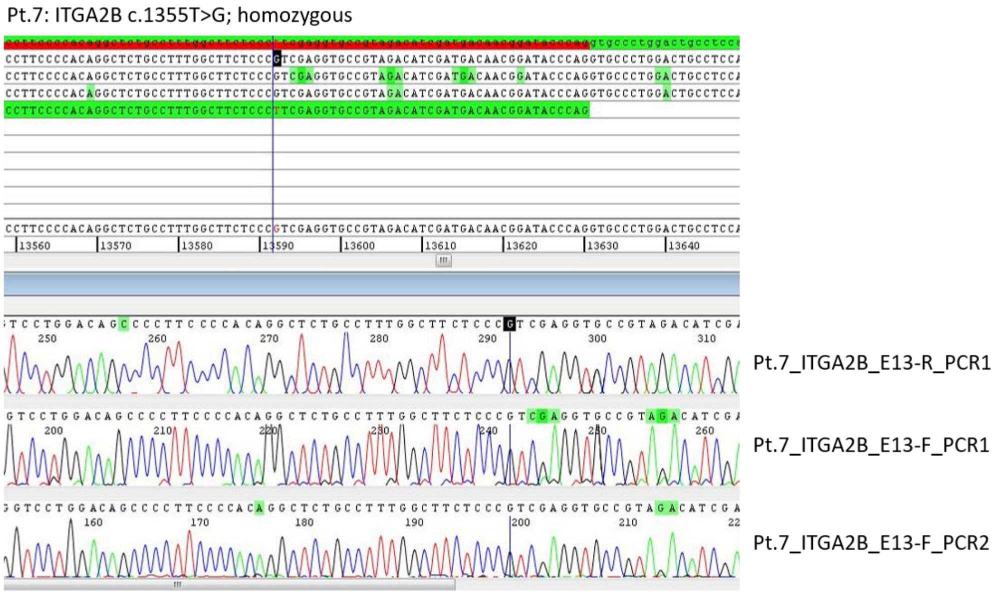

Figure S7: Chromatograms of Pt.7 showing the homozygous non-synonymous variant c.1355T>G (p.Leu452Arg) in *ITGA2B*

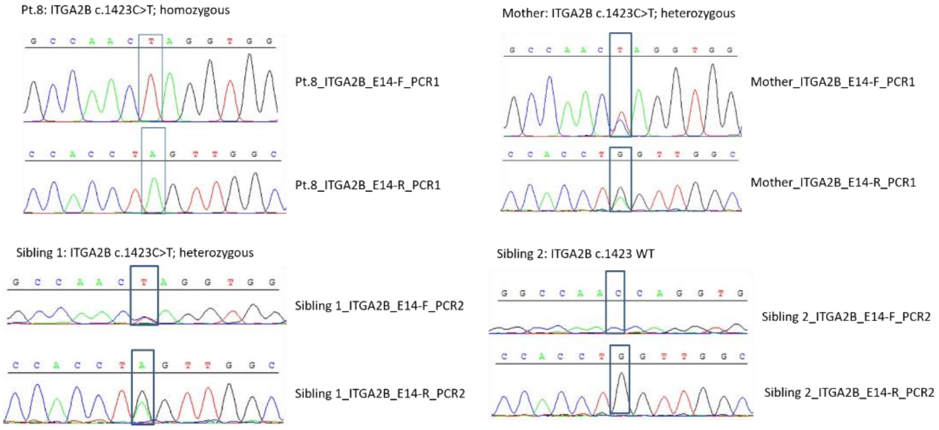

Figure S8: Family genotyping; chromatograms for Pt.8 showing the homozygous nonsense variant c.1423C>T (p.Gln475*) in *ITGA2B.* The mother and sibling 1 are heterozygous. Sibling 2 presented with wild type sequence.

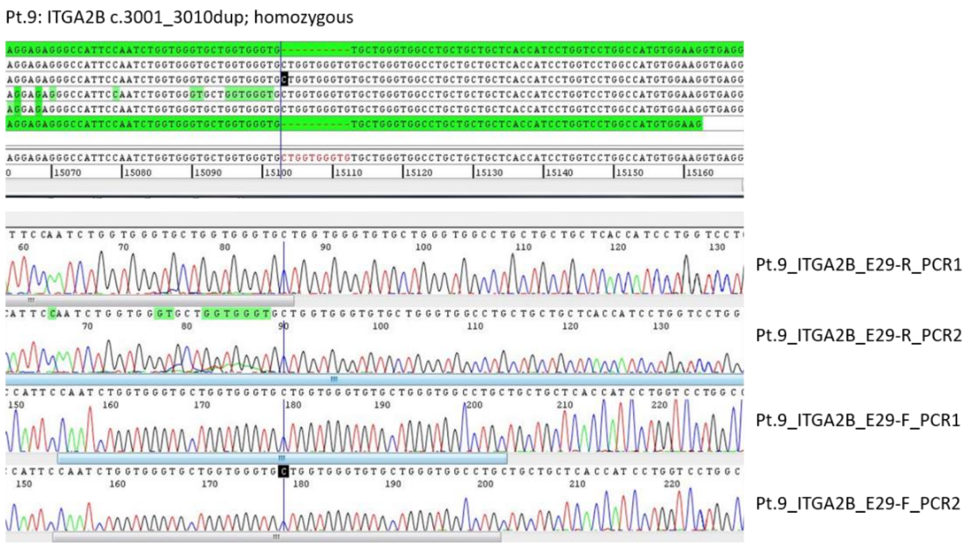

Figure S9: Chromatograms of Pt.9 showing the homozygous duplication c.3001_3010dup (p.Val1004Alafs*35) in *ITGA2B*

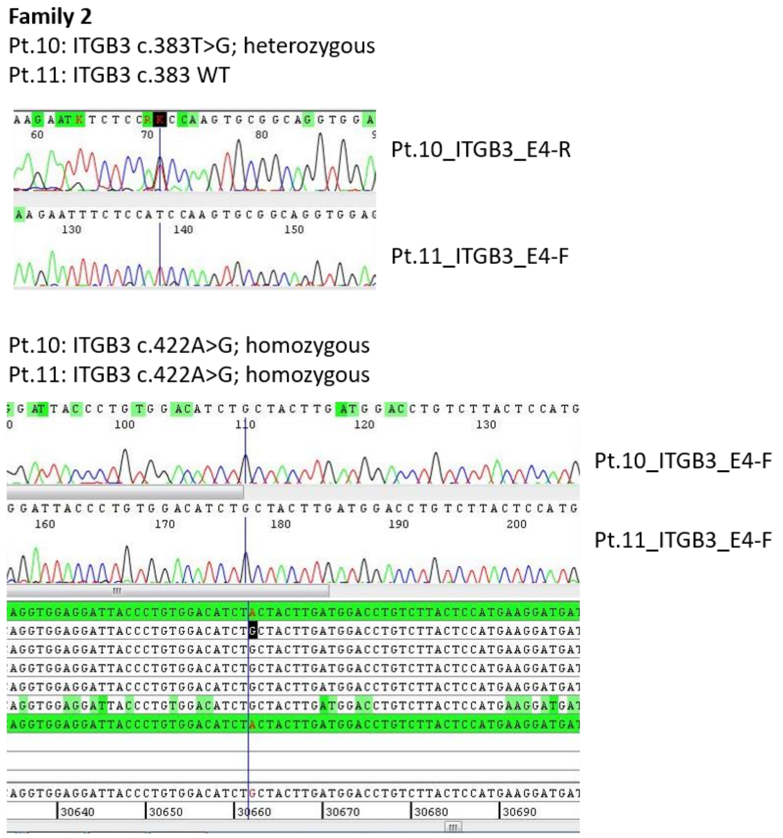

Figure S10: Chromatograms of Pt.10 and Pt.11 showing the heterozygous non-synonymous variant c.383T>G (p.Ile128Ser) in *ITGB3* in Pt.10 and wild type sequence in Pt.11. Both siblings are homozygous carrier of c.422A>G (Tyr141Cys).

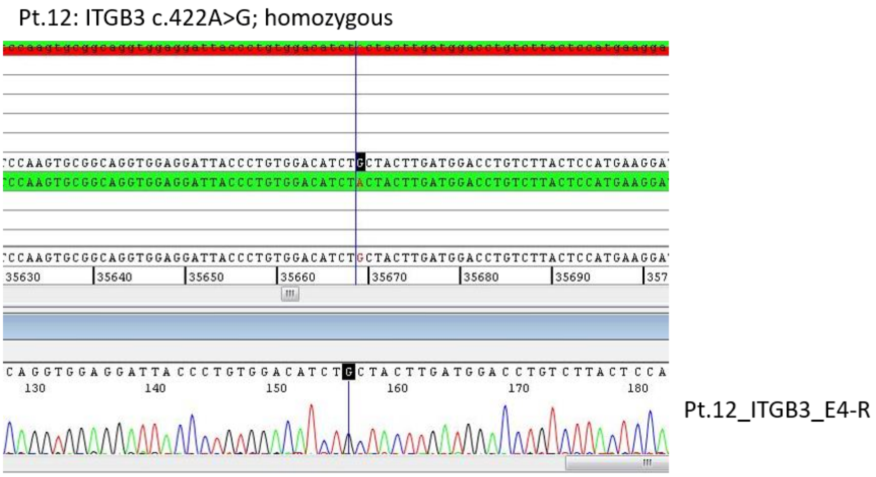

Figure S11: Chromatogram of Pt.12 showing the homozygous non-synonymous variant c.422A>G (Tyr141Cys) in *ITGB3*.

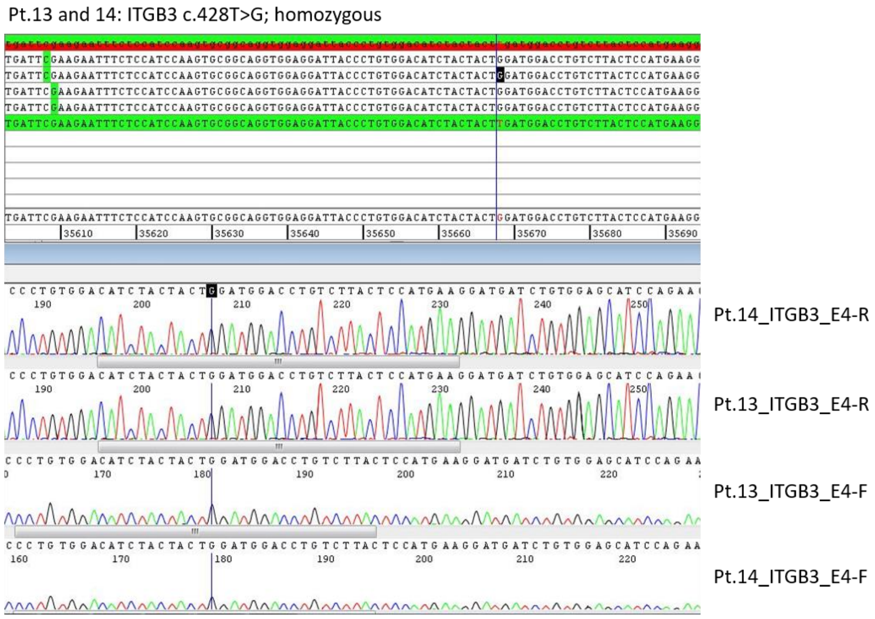

Figure S12: Chromatograms of Pt.13 and Pt.14 (family 3) showing the homozygous non-synonymous variant c.428T>G (p.Leu143Trp) in *ITGB3*.

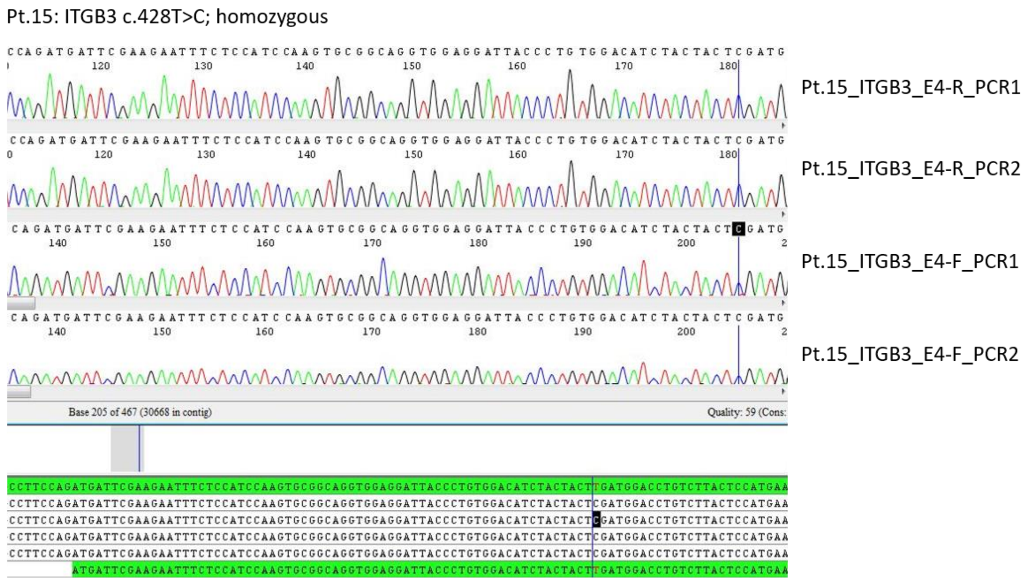

Figure S13: Chromatograms of Pt.15 showing the homozygous non-synonymous variant c.428T>C (p.Leu143Ser) in *ITGB3*.

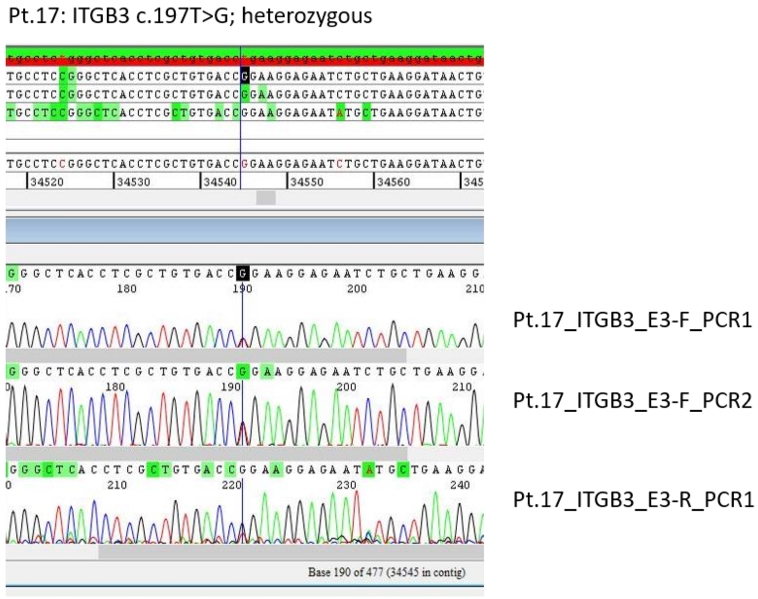

Figure S14: Chromatograms of Pt.17 showing the homozygous non-synonymous variant c.197T>G (p.Leu66Arg) in *ITGB3*.
